# Cardioprotective Effects of SAR Through Attenuating Cardiac-Specific Markers, Inflammatory Markers, Oxidative Stress, and Anxiety in Rats Challenged with 5-Fluorouracil

**DOI:** 10.3390/jox15040130

**Published:** 2025-08-10

**Authors:** Roza Haroon Rasheed, Tavga Ahmed Aziz

**Affiliations:** Department of Pharmacology and Toxicology, College of Pharmacy, University of Sulaimani, Sulaimani 46004, Kurdistan Region, Iraq; roza.rasheed@univsul.edu.iq

**Keywords:** SAR, 5-FU, cardiac-specific biomarkers, anti-inflammatory activity, antioxidant effect, anxiolytic effect

## Abstract

This study aimed to evaluate the cardioprotective effects of two different doses of saroglitazar (SAR) in an animal model of cardiotoxicity induced by 5-fluorouracil (5-FU). Thirty-five rats were randomly allocated into five groups: the negative control, which received distilled water; the 5-FU (150 mg/kg as I.P.) group; the N-acetylcysteine (100 mg/kg) group; and the SAR (0.5 and 5 mg/kg) groups. The last three groups received 5-FU on day 10 along with their treatment. An open field test was performed at zero-time and at the end of the study. On day eleven the animals were euthanized and blood samples were used for measuring troponin I, CK-MB, natriuretic peptide, lipid profile, LDH, ALT, AST, CRP, ESR, TNF-α, IL1β, MDA, and total antioxidant capacity (TAOC). Cardiac tissues were sent for histopathological examination. The study revealed that 5-FU elevated the levels of cardiac-specific and injury-related biomarkers, inflammatory and oxidative stress markers, and that the use of SAR, particularly the high dose, decreased all the cardiac- and other injury-related biomarkers as well as attenuating inflammatory and oxidative stress biomarkers. SAR-treated groups exhibited a significant increase in locomotor activity and a decrease in anxiety-like behavior, indicated by a reduction in time spent in one square and an increase in total movement time. Additionally, the histopathological findings greatly supported the biochemical results evidenced by stopping the detrimental effects caused by 5-FU through structural and functional alterations of cardiac tissues manifested as ameliorating congestion, inflammation, degeneration, arterial wall thinning, and endothelial loss. The dual-acting PPAR agonist SAR demonstrated cardiac protection activity, particularly the high dose, by attenuating cardiac-specific and nonspecific injury biomarkers along with anti-inflammatory and antioxidant activities and attenuated anxiety induced by 5-FU. These findings render SAR a promising candidate to be tested in clinical trials. Further studies are warranted with other cardiotoxicants to confirm these findings.

## 1. Introduction

In contemporary medicine, pharmacological management is essential, especially when it comes to the treatment of cancer. Nevertheless, several therapeutic medications have adverse reactions, with cardiotoxicity being a major concern. A variety of cardiovascular problems, such as myocardial ischemia, heart failure, arrhythmias, and pericarditis, are included in drug-induced cardiotoxicity. These conditions can impair patient outcomes and restrict available treatments [1].

Cancer chemotherapeutic agents, despite their effectiveness in terms of reducing tumor growth, are among the most well-known causes of cardiotoxicity [2]. The dose-dependent cardiotoxic effects of anthracyclines, such as doxorubicin, are well-established and linked to mitochondrial dysfunction and oxidative stress [3,4]. Similarly, drugs such as trastuzumab cause heart failure by interfering with the HER2 signaling pathways that are essential for cardiomyocyte survival [5]. Even though these mechanisms have been thoroughly investigated, there is still rising worry about the cardiotoxicity of other substances, such as antimetabolites like 5-fluorouracil (5-FU) [5,6].

For a variety of solid tumors, such as colorectal, breast, and head and neck malignancies, 5-FU is a commonly utilized chemotherapeutic drug. Its effectiveness stems from its capacity to block thymidylate synthase, which stops DNA synthesis in cells that divide quickly [7]. However, 5-FU has been linked to cardiotoxic side effects, such as myocardial ischemia, chest discomfort, and, in extreme situations, acute myocardial infarction. Although the precise mechanisms are still unknown, these effects are believed to be caused by oxidative stress, endothelial damage, and coronary vasospasm [8,9].

The clinical relevance of cardiotoxicity highlights the necessity of approaches to lessen its effects. Cardiotoxicity endangers patient survival and also requires pauses or cessation of essential treatments. Consequently, the application of cardioprotective agents has arisen as a hopeful strategy [10]. Medications like calcium channel blockers, beta-blockers, and angiotensin-converting enzyme (ACE) inhibitors, trimetazidine, statins, N-acetylcysteine (NAC), and dexrazoxane have demonstrated promise in lowering both the frequency and intensity of cardiotoxic effects linked to cancer therapies [11,12]. In addition, current studies seek to discover new cardioprotective agents and improve risk assessment methods to tailor treatments and boost patient safety.

Peroxisome proliferator-activated receptors (PPARs) are nuclear receptors responsible for controlling gene expression related to metabolism, inflammation, and cellular activities. PPAR-γ, such as thiazolidinediones, has a significant role in cardiovascular conditions. However, extensive research is still required to determine its role in the circulatory system. PPAR-α/γ agonists provide advantages in diabetes and atherosclerosis, recognized risk factors linked with cardiovascular disease. They are advantageous as treatment option agents leading to enhanced insulin sensitivity, decreased blood glucose levels, and reduced inflammation [13,14].

Saroglitazar (SAR) is an innovative dual agonist of peroxisome proliferator-activated receptors (PPARs) that mainly acts on PPAR-α and shows moderate effects on PPAR-γ. Created to address dyslipidemia and diabetic dyslipidemia, SAR has exhibited encouraging protective benefits for the cardiovascular system by enhancing lipid metabolism, lowering inflammation, and alleviating metabolic irregularities [15,16]. Compared to conventional PPAR agonists, SAR exhibits a good safety profile with a low risk of fluid retention or adverse cardiovascular events [17].

Given Saroglitazar’s dual PPAR-α/γ agonist activity and its established benefits in improving lipid metabolism, reducing inflammation, and promoting cardiovascular health, the present study aimed to investigate the potential cardioprotective effects of Saroglitazar in a rat model of 5-fluorouracil (5-FU)-induced cardiotoxicity.

## 2. Methodology

Thirty-five male Albino Wistar rats, weighing (240–280 g), were utilized in the current study. The animals were kept for two weeks for acclimatization before starting the study; they were kept in well-ventilated cages with free access to water and pellets. The environment of the animal house was standard in terms of temperature (25 ± 2 °C), humidity (55 ± 5%), and the 12 h day–night cycle. The study design and protocols were approved by the Ethical Committee of the College of Pharmacy (Certificate no. PH 145-24 on 28 November 2024), and the study was conducted in accordance with ARRIVE guidelines. The doses of saroglitazar [18], 5-FU [7], and N-acetylcysteine [19], and the duration of the study [18,20,21], were chosen based on previous studies. All animals were divided into five groups randomly as follows:

1. Negative control (NC) group: They were treated orally with 1 mL of distilled water (D.W.) by oral feeding tube for 10 days.
2. 5-Florouracil (5-FU) group: They were treated the same as the negative control group, but on day 10 they received (150 mg/kg) as a single intraperitoneal (I.P.) dose.
3. N-acetyl cysteine (NAC) group: Treated with 100 mg/kg NAC daily orally for 10 days. This group’s treatment is a standard cardioprotective agent known for its well-established antioxidant, anti-inflammatory, and cytoprotective properties [22].
4. Low dose of SAR group: Treated with 0.5 mg/kg SAR orally for 10 days.
5. High dose of SAR: Treated with 5 mg/kg SAR orally for 10 days.

The last three groups received a single dose of 5-FU on day 10. On day eleven, all the treatment groups were euthanized, and blood samples were collected for biochemical tests and the heart was excised and kept in 10% formalin for histopathological examination. Histopathological evaluations were performed by an experienced histopathologist who was blinded to the group allocations throughout the analysis to reduce the potential for observer bias. The rats were weighed prior to the initiation of treatment and on scarification day, utilizing a weighing scale.

### 2.1. Biochemical Tests

The blood was collected from the animals by cardiac puncture and used to assess Troponin I, MB-CPK, Natriuretic peptide, IL-1β, TNF-α, MDA, and TAOC, which were measured using ELISA kits (Bioassay Technology Laboratory, Shanghai, China); total lipid profile, AST, ALT, LDH, ALP, CRP, ESR, and Creatinine were measured using ready-made kits (Randox, London, UK).

### 2.2. Determination of Atherogenic Indices

The atherogenic indices were calculated as follows [23,24]:

Atherogenic Coefficient: (Total cholesterol − HDL)/HDL

Atherogenic Index in Plasma (AIP) = Log (TG/HDL)

Cardiac Risk Ratio (CRR) = Total cholesterol/HDL

### 2.3. Evaluation of Locomotor Behavior and Anxiety Test in Rats

#### Open Field Test

This experiment was based on a modification of two studies [25,26]. An open field test (OFT) was conducted to evaluate exploratory behavior and anxiety levels in 35 albino male Sprague–Dawley rats, systematically categorized into five groups of seven rats each. The experimental setup comprised a 100 cm × 100 cm arena with 40 cm high walls, distinctly marked to facilitate the recording of line crossings and zone occupancy. Each rat was separately positioned in the center of the arena, permitting unrestricted exploration for a defined length of 5 min. Behavioral observations were systematically documented using an overhead camera connected to motion-tracking software, allowing precise data collection.

The primary behavioral parameters assessed were the number of line crossings to gauge overall locomotion and exploratory motivation, occurrences of rearing (both wall-supported and free-standing) as measures of vertical exploration, and the frequency of grooming, urination, and defecation as signs of stress. The duration spent in the center compared to the margins of the arena was recorded, with a tendency for the margins to signify elevated anxiety levels. The total movement time and the duration spent in a particular grid square were recorded to evaluate general mobility and localized anxiety triggers, respectively. This thorough investigation sought to elucidate the effects of various therapies on anxiety-related behaviors and general exploratory activities in a controlled environment, thereby offering significant insights into the psychopharmacological effects of these treatments in a preclinical paradigm.

### 2.4. Histopathological Protocol

The complete heart was removed after animal scarification, cut into two slices, and cleaned of blood and fatty tissue from the epicardium. After being trapped in plastic tissue cassettes, heart slices were fixed for at least 48 h in 10% neutral buffered formalin. Heart tissues were dehydrated using four sequential adjustments of increasing ethanol alcohol concentrations: 60%, 70%, 90%, and 100%. After being cleansed with xylene, the tissue samples were embedded in paraffin. A semi-automated microtome (Danaher Corporation, Nussloch, Germany) was used to cut and segment the tissue-wax blocks to 5 µm. Tissue sections were also mounted and fixed on glass slides using a hot-water bath, and they were dried for 30 min on a hot plate. Slices of the heart were then submerged in two xylene changes for five minutes each to deparaffinize them. Four decreasing ethanol concentrations (100%, 90%, 70%, and 60%) were then used to rehydrate them for five minutes each. The tissue sections were stained using Harris’s hematoxylin and eosin (H&E) method. Using the mounting solution Distrene Plasticizer Xylene (DPX), the sections were finally washed with xylene, dried, and mounted with glassy cover slips.

### 2.5. Semiquantitative Histomorphometry Analysis of Myocardial Toxicity

The cardiac tissues were examined by a light microscope image analyzer set to high-power 400× magnifications for semi-quantitative morphometric analysis. The tissue samples were viewed under a light microscope (Olympus BX51, Olympus Corporation, Tokyo, Japan) with an AmScope 3.7 image analyzer (MU300, 2019) attached to a digital camera. To assess myocardial toxicity, various parameters were quantified from stained myocardial tissues and statistically compared to determine significant differences between the model and different treatment groups. These parameters included capillary congestion, inflammatory cell infiltration, endothelial cell loss in myocardial coronary arteries, thinning of the arterial wall, and areas of myocardial degeneration.

The number of congested capillaries was counted in all fields displaying capillary congestion across the examined tissues. This analysis was performed for all slides in each group under 100× magnification. The mean values for each group were then calculated. The percentage of the mean values was compared to identify significant differences. Additionally, grading and scoring were performed to further evaluate the severity of congestion.

The quantification of inflammation, arterial wall thinning, endothelial loss, and areas of tissue degeneration were assessed at a 400× magnification. For the quantification of inflammation, visible inflammatory cells were counted across all slides in each group. The mean values were obtained and statistically compared to detect significant differences. Additionally, grading and scoring were performed to assess the severity of inflammation.

For the intramyocardial coronary artery, two parameters were quantified and evaluated: endothelial cell loss and arterial wall thinning. To assess endothelial cell loss, the nuclei of flattened endothelial cells were counted in all slides of the normal group, and their mean value was established as the reference standard. For each treated group, endothelial cells were counted across all slides, and the mean values were calculated. The percentage of endothelial cell loss was then determined using the following formula:

%Endothelial Cell Loss = (Total endothelial cells in normal artery − Remaining endothelial cells in treated groups ÷ Total endothelial cells in normal artery) × 100

This approach allowed for the statistical comparison of endothelial damage across different groups.

To determine the percentage of arterial wall thinning, the thickness of the intramyocardial artery wall was measured in microns at ten different points along the circular arterial circumference for each slide in the normal group. The mean values were calculated to establish a standard reference. Similarly, the arterial wall thickness was measured along the circular arterial circumference for all slides in each treated group, and the mean values were determined. These means were then used to calculate the percentage of arterial wall thinning.

The percentage of wall thinning was calculated using the following formula:

%Arterial Wall Thinning = (Normal Thickness − Thinned Thickness ÷ Normal Thickness) × 100

To quantify the degenerated area, all histopathological fields displaying myocardial necrosis or intracytoplasmic vacuoles—indicators of degeneration—were analyzed. The total visible area under a 400× magnification was measured in square microns (µm^2^) for each section, along with the area of degeneration within the same section. This process was repeated across ten different areas per slide for each group, and the mean values were calculated. The percentage of degenerated area was determined using the following formula:

%Degenerated Area = (Degenerated Area ÷ Total Area) × 100

Scoring and grading were performed according to the criteria presented in Table 1. A *p*-value of less than 0.05 was considered statistically significant.

### 2.6. Statistical Analysis

Statistical analysis was performed using GraphPad Prism 8. The normality of the data distribution was assessed using the Shapiro–Wilk test. Data that followed a normal distribution were expressed as mean ± standard deviation (S.D.) and analyzed using one-way ANOVA followed by Tukey’s multiple comparisons test. For all analyses, a *p*-value of less than 0.05 was considered statistically significant.

## 3. Results

### 3.1. Impact of Different Doses of SAR on Cardiac-Specific Biomarkers, and Cardiac Indices

**Figure 1:**
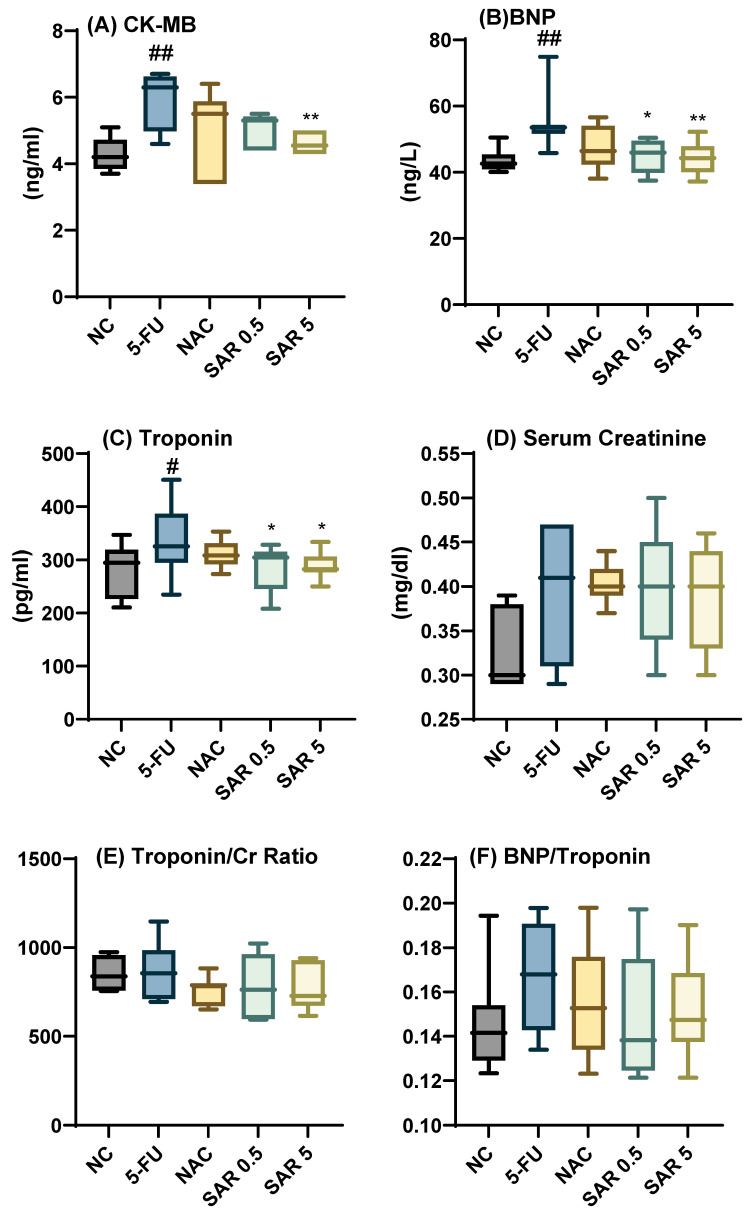

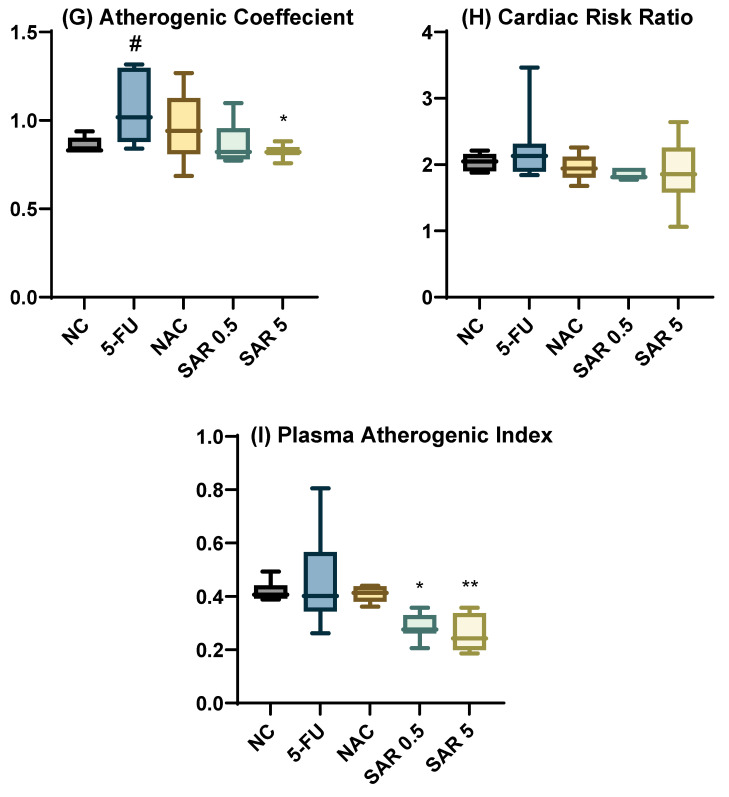
Impact of different doses of SAR on cardiac-specific markers and atherogenic indices: (**A**) CK-MB, (**B**) BNP, (**C**) Troponin, (**D**) Serum Creatinine, (**E**) Troponin/Cr ratio, and (**F**) BNP/Troponin ratio, (**G**) Atherogenic coefficient, (**H**) cardiac risk ratio, (**I**) Plasma Atherogenic Index. Values are presented as mean ± S.D (n = 7). Based on one-way ANOVA followed by Tukey’s post hoc test, values with (#) indicate that 5-FU significantly differs from the NC group, (# *p*-value < 0.5 and ## *p*-value < 0.01); (*) indicates a statistically significant difference with the 5-FU group (* *p*-value < 0.05 and ** *p*-value < 0.01).

A significant elevation in the level of CK-MB was observed in the 5-FU group compared to the NC group (*p*-value = 0.003). The elevation produced by 5-FU was attenuated significantly by the high dose of SAR. In contrast, the reduction made by each of the NAC and SAR (0.5 mg/kg) groups did not reach a significant level (*p*-value > 0.05). The level of BNP was increased significantly in the 5-FU group when compared to the NC group (*p*-value = 0.005); both low and high doses of SAR significantly decreased the level, (*p*-value = 0.016), and (*p*-value = 0.008), respectively. Meanwhile, NAC resulted in a non-significant attenuation, (*p*-value > 0.05). The administration of 5-FU resulted in a significant elevation in the level of troponin in comparison with the NC group, (*p*-value = 0.02). No significant reduction was seen with the NAC group, (*p*-value > 0.05); meanwhile, both low- and high-dose SAR groups significantly ameliorated troponin levels when compared to the 5-FU group, (*p*-value = 0.03) and (*p*-value = 0.04), respectively. A non-significant reduction was observed in serum creatinine levels and the troponin-to-S.Cr and BNP-to-troponin ratios in all the treatment groups, (*p*-value > 0.05) when compared with the 5-FU group (Figure 1A–F). Regarding cardiac atherogenicity indices, 5-FU significantly increased the atherogenic coefficient, (*p*-value = 0.03), and the use of a high dose of SAR resulted in a significant reduction in the atherogenic coefficient, (*p*-value = 0.02), and AIP, (*p*-value = 0.03), with a non-significant reduction in cardiac risk ratio. Meanwhile, the low dose of SAR attenuated the AIP only, (*p*-value = 0.03) (Figure 1G–I).

### 3.2. Impact of Different Doses of SAR on Weight Change, AST, ALT, LDH, and ALP

5-Fluorouracil did not affect the delta total body weight (ΔW) compared to the control group. Meanwhile, the NAC and high-dose SAR groups produced a non-significant reduction in ΔW (*p* ˃ 0.05) compared to the 5-FU group. Meanwhile, the low-dose SAR group achieved a significant decrease in ΔW (*p* < 0.05) in comparison with all the other groups.

AST levels were elevated significantly in the 5-FU group, (*p*-value = 0.0003), and in all the treatment groups; NAC and low and high doses of SAR ameliorated AST levels compared with the NC group, (*p*-value = 0.03), (*p*-value = 0.04), and (*p*-value = 0.02), respectively. Additionally, the ALT level decreased significantly in the 5-FU group, (*p*-value = 0.04), and only the high-dose SAR group significantly attenuated its level (*p*-value = 0.005), compared to the 5-FU group. No significant changes were observed in AST/ALT ratios except for the low dose of SAR compared to the 5-FU group, (*p*-value < 0.05). Regarding the effect on ALP, 5-FU raised the level significantly (*p*-value = 0.013) compared to the NC group and each of NAC, SAR 0.5 mg/kg, and SAR 5 mg/kg attenuated the level of ALP significantly compared to the 5-FU group, (*p*-value = 0.04), (*p*-value = 0.006), and (*p*-value = 0.001), respectively. LDH levels increased significantly in the 5-FU group (*p*-value = 0.0009) compared to the NC group and all the treatment groups decreased their level; however, only the NAC group reached a significant level when compared to the 5-FU group (*p*-value = 0.0017), (Table 2).

### 3.3. Impact of Different Doses of SAR on Inflammatory and Oxidative Stress Biomarkers

**Figure 2:**
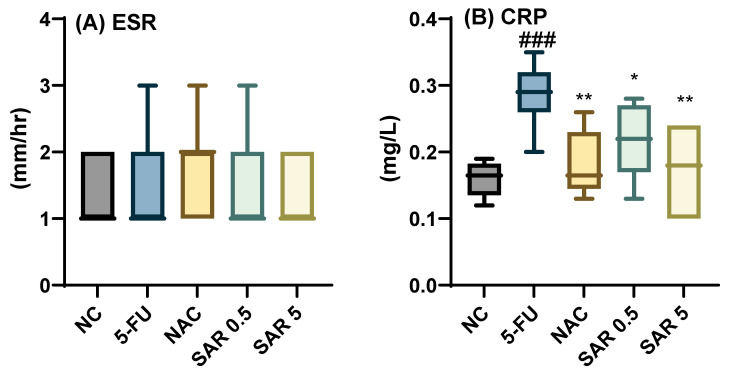

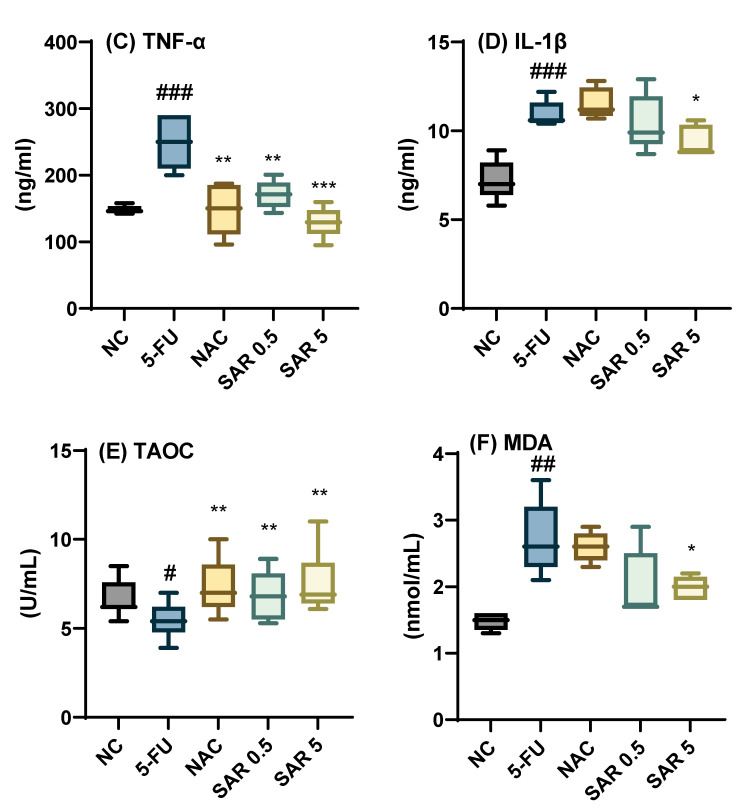
Impact of different doses of SAR on atherogenic indices: (**A**) ESR, (**B**) CRP, (**C**) TNF-α, (**D**) IL-1β, (**E**) TAOC, and (**F**) MDA. Based on one-way ANOVA followed by Tukey’s post hoc test, values with (#) indicate that 5-FU significantly differs from the NC group, (# *p*-value < 0.5, ## *p*-value < 0.01, and ### *p*-value < 0.001); (*) indicates a statistically significant difference with the 5-FU group (* *p*-value < 0.05, ** *p*-value < 0.01, and *** *p*-value < 0.001).

Treatment with 5-FU increased ESR level; however, it did not reach a significant level and all the treatment groups non-significantly attenuated its level (*p*-value > 0.05). Meanwhile, CRP was increased significantly by 5-FU, (*p*-value = 0.0003) compared to NC, and all the treatment groups, NAC (*p*-value = 0.004), SAR 0.5 mg/kg (*p*-value = 0.03), and SAR 5 mg/kg (*p*-value = 0.004), successfully ameliorated the level of CRP compared to 5-FU. Regarding the IL-1β level, the use of 5-FU significantly elevated it compared to the NC group (*p*-value = 0.0001), and only the high dose of SAR significantly attenuated its level compared to the 5-FU group (*p*-value = 0.017). Additionally, the level of TNF-α increased significantly in the 5-FU group compared to the NC, (*p*-value = 0.0006), and treatment with NAC, (*p*-value = 0.003), SAR 0.5 mg/kg, (*p*-value = 0.004), and SAR 5 mg/kg, (*p*-value = 0.0004), resulted in significant attenuation compared to the 5-FU group, (Figure 2A–D).

The level of TAOC was mitigated significantly by 5-FU (*p*-value = 0.03) compared to the NC group, and this level was elevated significantly by each of NAC (*p*-value = 0.001), SAR 0.5 mg/kg (*p*-value = 0.008), and SAR 5 mg/kg (*p*-value = 0.002). Furthermore, the MDA level was increased significantly by 5-FU, (*p*-value = 0.001), and only the high dose of SAR was able to attenuate its level compared to 5-FU, (*p*-value = 0.02) (Figure 2E,F).

### 3.4. Impact of Different Doses of SAR on Anxiety

#### 3.4.1. Number of Lines Crossed, Rearing, Walling, Grooming, and Frequencies of Urination and Defecation

At zero-time, no significant differences were observed in the number of crossed lines across all treatment groups. On day 11, regarding the 5-FU-treated group, there was a significant decrease (*p*-value < 0.01) in the number of lines crossed compared to the control group. However, in the N-acetylcysteine-receiving group, a non-significant increase (*p*-value ˃ 0.05) in the number of crossed lines was observed compared with the 5-FU group. Meanwhile, a significant increase (*p*-value < 0.05) in the number of lines crossed was observed in the low-dose SAR-treated group compared to the 5-FU group. Also, the number of f lines crossed was significantly higher (*p*-value < 0.05) in the high-dose SAR-treated group compared to the 5-FU group.

At zero-time, all the groups had a non-significant variation (*p*-value ˃ 0.05). Concerning day 11, a significant decrease (*p*-value < 0.05) was observed in rearing in the 5-FU-treated group compared with the control group, while the n-acetylcysteine produced a non-significant rise (*p*-value ˃ 0.05) in rearing compared to the 5-FU-treated group. Moreover, the SAR in the low-dose SAR-treated group resulted in a significant increase (*p*-value < 0.01) in the rearing rate compared to the 5-FU-treated group. Similarly, the SAR in the high-dose SAR-treated group produced a significant elevation (*p*-value < 0.05) in the rearing rate compared with the 5-FU group.

At zero-time, no significant differences were observed in walling rate among all groups treated. Meanwhile, on the 11th day, a significant reduction (*p*-value < 0.05) was noticed in the walling rate in the 5-FU-receiving group compared with the negative control group. A non-significant variation (*p*-value ˃ 0.05) was observed in the groups treated with n-acetylcysteine, a low dose of SAR, and a high dose of SAR, in comparison to the 5-FU group.

The present study revealed that the 5-FU-treated group exhibited a non-significant decrease (*p*-value ˃ 0.05) in grooming rate in comparison with the control group. Meanwhile, the n-acetylcysteine in the NAC group resulted in a significant increase (*p*-value < 0.01) in grooming rates as compared with the 5-FU group. Meanwhile, the SAR resulted in a non-significant increase (*p*-value ˃ 0.05) in grooming frequencies in both the low-dose and high-dose groups as compared with the 5-FU group. Regarding day 11, the 5-FU resulted in a significant decrease (*p*-value < 0.05) in the rates of grooming in the 5-FU-treated group (PC group) when compared with the control group, while in the NAC group, the n-acetylcysteine produced a non-significant increase (*p*-value ˃ 0.05) in grooming frequency as compared with the 5-FU group, and the use of SAR resulted in a non-significant increase (*p*-value ˃ 0.05) in the rates of grooming in both the low-dose and high-dose SAR-treated groups in comparison to the 5-FU group.

Regarding the frequency of urination, all treated groups showed a non-significant effect at the zero-time. While on day 11, the 5-FU-treated group exhibited a significant decrease (*p*-value < 0.05) in the frequency of urination when compared with the control group. However, the N-acetylcysteine-treated group showed no change in urination rates compared to the 5-FU group, while the low dose of SAR group showed a non-significant increase (*p*-value ˃ 0.05) compared to the 5-FU group. Additionally, in the high-dose SAR-treated group, SAR produced a significant increase (*p*-value < 0.05) in the frequency of urination as compared with the 5-FU group.

**Figure 3:**
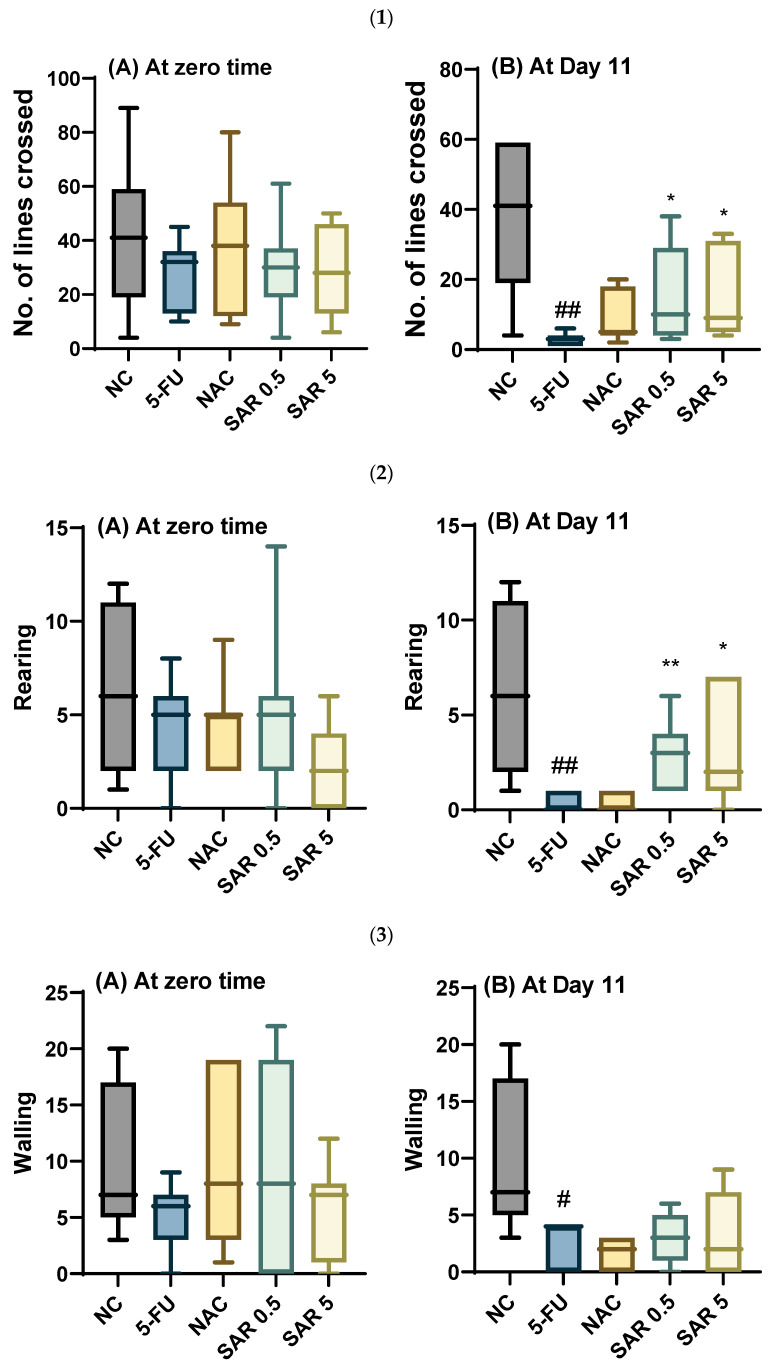

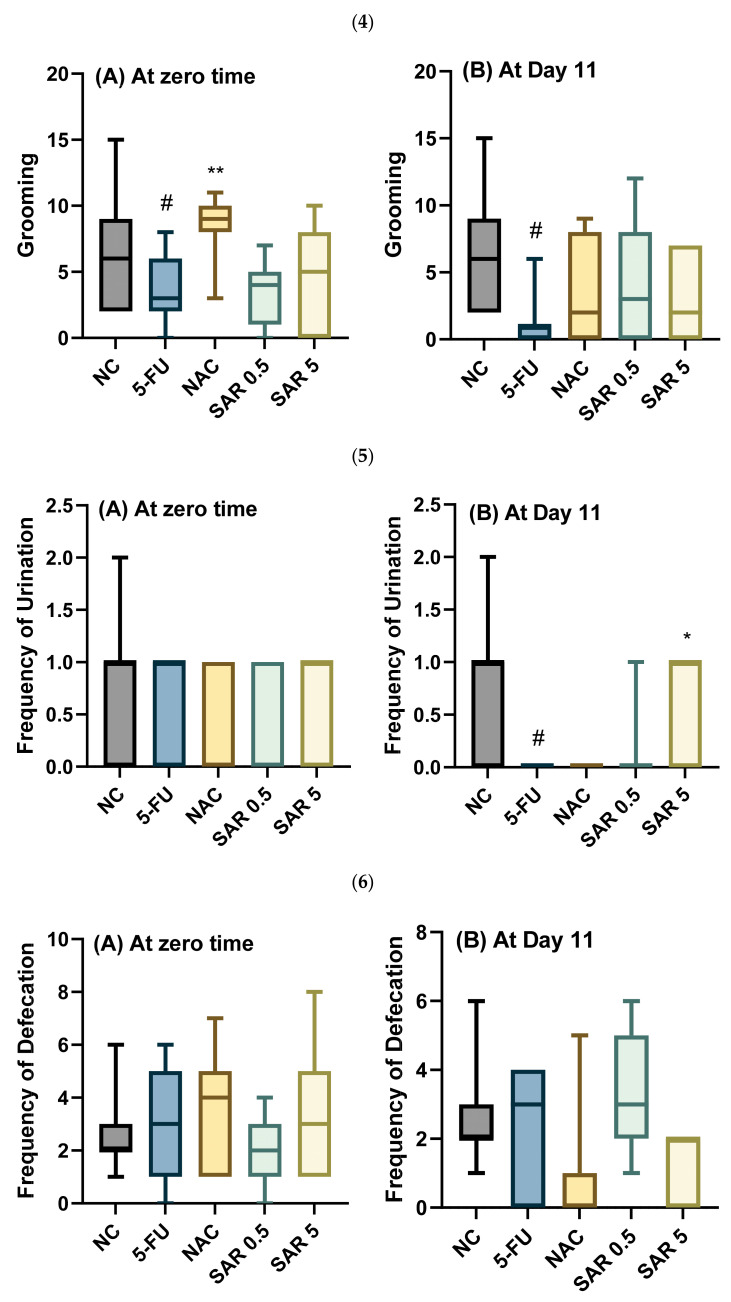
Impact of SAR on behavioral test: (**1**) Number of lines crossed (**A**) at zero-time and (**B**) at Day 11. (**2**) Rearing (**A**) at zero-time and (**B**) at Day 11. (**3**) Walling (**A**) at zero-time and (**B**) at Day 11. (**4**) Grooming (**A**) at zero-time and (**B**) at Day 11. (**5**) Urination (**A**) at zero-time and (**B**) at Day 11. (**6**) Defecation (**A**) at zero-time and (**B**) at day 11. Values are presented as mean ± S.D (n = 7). Based on one-way ANOVA followed by Tukey’s post hoc test, values with (#) indicate that 5-FU significantly differs from the NC group, (# *p*-value < 0.5, and ## *p*-value < 0.01); (*) indicates a statistically significant difference with the 5-FU group (* *p*-value < 0.05 and ** *p*-value < 0.01).

Moreover, all the groups showed non-significant differences (*p*-value ˃ 0.05) in the frequency of defecation. Similarly, on day 11, there were also no significant differences (*p*-value ˃ 0.05) in the rates of defecation for all treated groups (Figure 3).

#### 3.4.2. Time Spent in the Center, Time Spent in the Margins, Total Time of Movement, and Total Time Spent in One Square

At zero-time, all the groups treated showed a non-significant difference (*p*-value ˃ 0.05) in the time spent in the center. Concerning day 11, 5-FU resulted in a significant decrease (*p*-value < 0.01) in the time spent in the center in the 5-FU-treated group when compared with the control group. Meanwhile, the NAC-receiving group produced a non-significant drop (*p*-value ˃ 0.05) in the time spent in the center compared to the 5-FU group. Moreover, a non-significant (*p*-value ˃ 0.05) decrease was observed in the low-dose SAR-treated group in comparison to the 5-FU group, while a significant (*p*-value < 0.05) rise was noticed in the high-dose SAR-treated group as compared to the 5-FU group.

Concerning the time spent at the margins, there was no significant difference in the duration spent at the margins among all treatment groups at zero-time. However, on day 11, the group treated with 5-FU exhibited a significant increase in the time spent at the margins (*p*-value < 0.01) compared to the control group. Conversely, the group treated with N-acetylcysteine showed an increase in margin time relative to the 5-FU group, though this change was not statistically significant (*p*-value > 0.05). Additionally, treatment with a low dose of SAR showed a non-significant increase (*p*-value > 0.05) in the duration spent at the margins compared to the 5-FU group. In contrast, a high dose of SAR significantly reduced (*p*-value < 0.05) the time spent at the margins relative to the 5-FU group.

At time-zero, there were no significant variations in total movement time among all treatment groups. On day 11, 5-FU caused a significant decrease (*p*-value < 0.0001) in the total time of movement in the 5-FU-treated group compared to the control group. Whereas, in the NAC-treated group, a non-significant elevation in the duration of movement was noticed as compared to the 5-FU group. Moreover, SAR produced a significant increase (*p*-value < 0.01) in the total time of movement in the low-dose SAR-treated group in comparison to the 5-FU group. Similarly, SAR also resulted in a significant rise in total time of movement as compared with the 5-FU-treated group.

**Figure 4:**
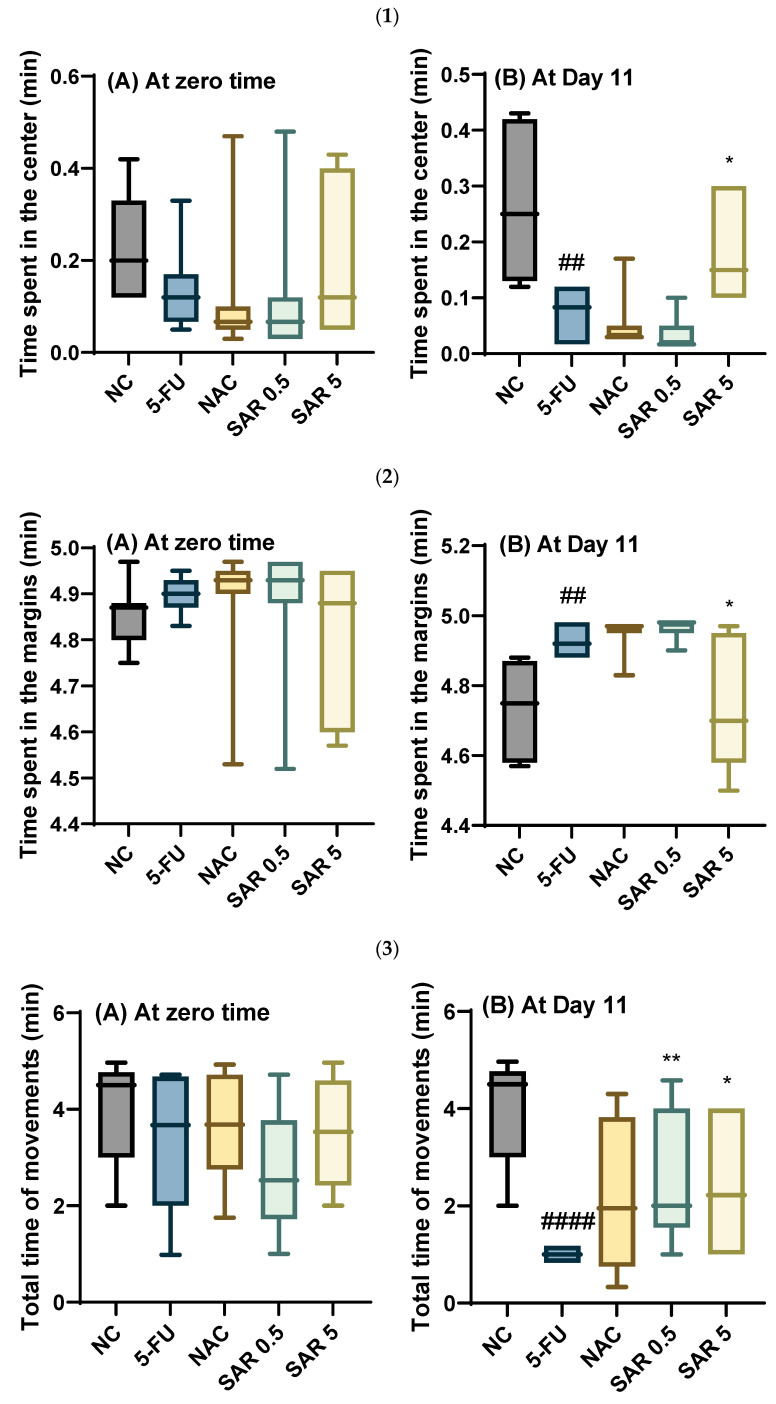

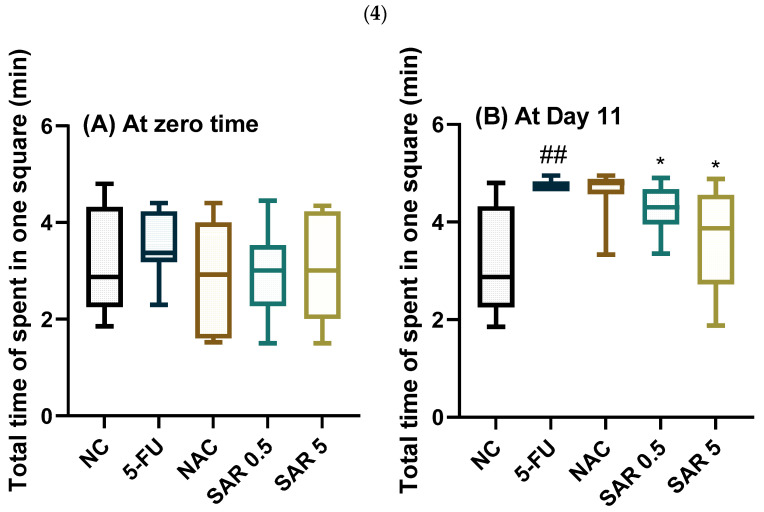
Impact of SAR on behavioral test: (**1**) time spent in the center (**A**) at zero-time and (**B**) at Day 11; (**2**) time spent at the margins (**A**) at zero-time and (**B**) at Day 11; (**3**) total time of movement (**A**) at zero-time and (**B**) at Day 11; (**4**) total time spent in one square (**A**) at zero-time and (**B**) at Day 11. Values are presented as mean ± S.D (n = 7). Based on one-way ANOVA followed by Tukey’s post hoc test, values with (#) indicate that 5-FU significantly differs from the NC group, (## *p*-value < 0.01, and #### *p*-value < 0.0001); (*) indicates a statistically significant difference with the 5-FU group (* *p*-value < 0.05, and ** *p*-value < 0.01).

Furthermore, there were no significant differences in the total time spent in one square between all the treated groups at zero-time. On day 11, a significant elevation (*p*-value < 0.01) was observed in the total time spent in one square in the 5-FU-treated group compared with the control group. Meanwhile, N-acetylcysteine produced a non-significant increase (*p*-value ˃ 0.05) in the duration spent in one square as compared with the 5-FU group. However, a low dose of SAR resulted in a significant reduction (*p*-value < 0.05) in total time spent in one square in comparison to the 5-FU-treated group, and the use of a high dose of SAR also produced a significant drop in the total time spent in one square compared with the 5-FU-treated group (Figure 4).

### 3.5. Histopathological Finding

**Figure 5:**
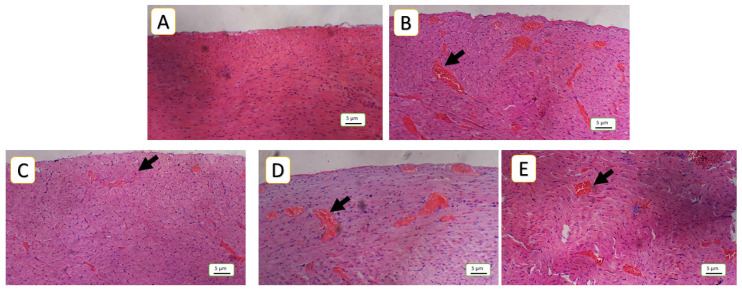
Histopathological assessment of cardiac tissue congestion: (**A**) represents negative control, normal cardiac tissue without any congested capillaries, corresponding to a congestion score of 0; (**B**) 5-FU (150 mg/kg) demonstrates severe congestion, characterized by a high number of congested capillaries corresponding to a congestion score of 3 as the model group, (**C**,**D**) in the NAC (100 mg/kg and SAR (0.5 mg/kg) groups; depict a moderate degree of congestion, corresponding to a congestion score of 2; (**E**) SAR (5 mg/Kg) shows a reduction in the number of congested capillaries, indicating mild congestion (score 1). Hematoxylin and eosin (H&E) staining, 100× magnification. Black arrows indicate congested capillaries.

**Figure 6:**
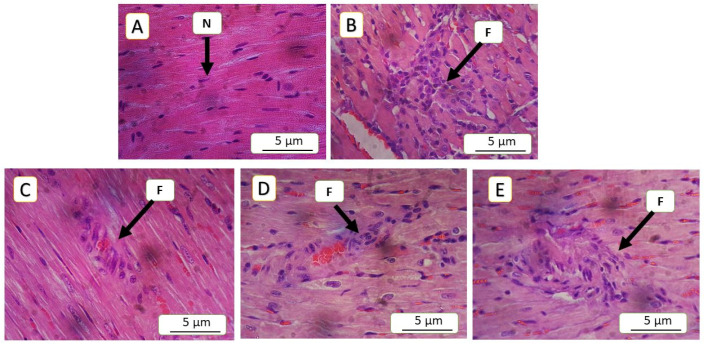
Histopathological assessment of chronic inflammatory cell infiltration in cardiac tissue: (**A**) represents the negative control group, showing no inflammatory cell infiltration (score 0), with clearly visible myocardial nuclei (N); (**B**) 5-FU (150 mg/kg demonstrates severe chronic inflammatory cell infiltration in the model group; (**C**,**D**) depict moderate chronic inflammatory cell infiltration, corresponding to a score of 2, in the NAC (100 mg/kg) and SAR (0.5 mg/kg)-treated and groups. (**E**) SAR (5 mg/kg) shows a reduction in inflammation to a mild degree (score 1) in the high-dose-treated group. The letter (F) indicates areas of inflammation, hematoxylin, and eosin (H&E) staining at 400× magnification.

**Figure 7:**
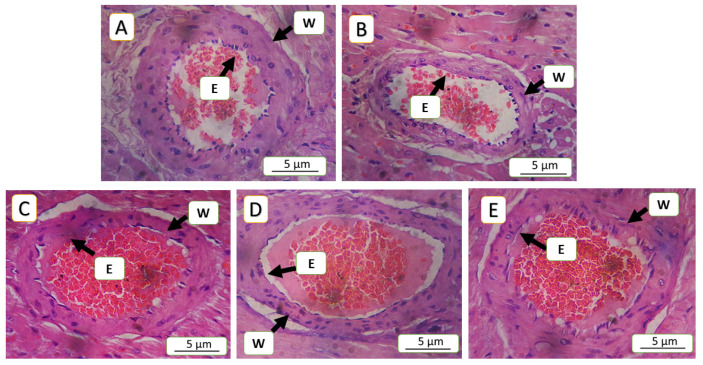
Histopathological evaluation of intramyocardial arteries, focusing on wall thickness and endothelial integrity: (**A**) represents a negative control as a normal artery with preserved histological architecture, normal wall thickness, and an intact endothelial lining, without significant pathological changes; (**B**) in the 5-FU (150 mg/kg) group, severe arterial wall thinning and extensive loss of endothelial lining are observed, corresponding to a score of 3; (**C**,**D**) in the NAC (100 mg/kg) and SAR (0.5 g/kg) groups, a moderate loss of endothelial cells and arterial wall thinning are noted, corresponding to a score of 2; (**E**) in the SAR (5 mg/kg) group, the arterial wall exhibits a mild degree of thinning and endothelial cell loss, corresponding to a score of 2, “W” denotes the arterial wall, and “E” represents endothelial cells, hematoxylin and eosin (H&E) staining at a 400× magnification.

**Figure 8:**
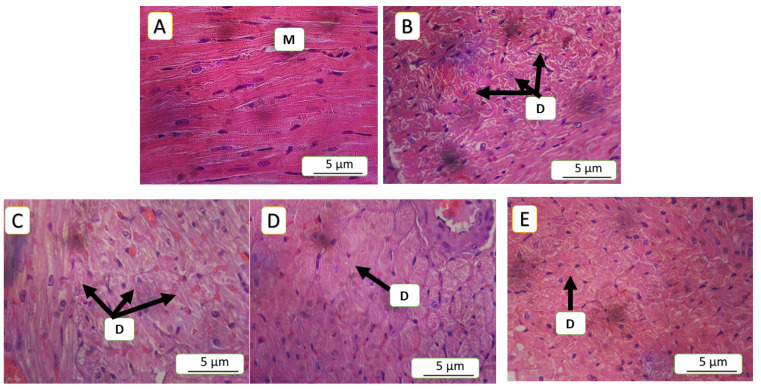
Myocardial tissue degeneration across different experimental groups: (**A**) depicts a negative control myocardial architecture with well-preserved myocardial cells, intact nuclei, and mitochondrial structures; (**B**) in the 5-FU (150 mg/kg) group, approximately 35% of the visible area is occupied by degenerated cells, characterized by necrotic cells and pyknotic nuclei, corresponding to a score of 3; (**C**,**D**) in the NAC (100 mg/kg)- and SAR (0.5 mg/kg)-treated groups, 20% and 21% of the visible area, respectively, show degeneration, corresponding to a score of 2 (moderate degeneration). (**E**) In the SAR (5 mg/kg)-treated group, 18% of the histopathologic area exhibits degeneration, corresponding to a score of 1; D indicates degenerated areas, hematoxylin and eosin (H&E) staining at a 400× magnification.

**Figure 9:**
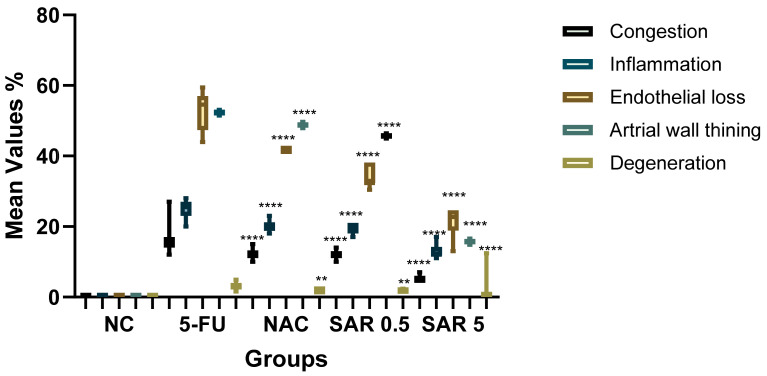
The line chart illustrates the myocardial toxicity scores of lesions across the examined groups. Values are presented as mean ± S.D (n = 7). Based on one-way ANOVA followed by Tukey’s post hoc test, values with (*) indicate a statistically significant difference with the 5-FU group (** *p*-value < 0.01, and **** *p*-value < 0.0001). NC: negative control; 5-FU: 5-Fluorouracil; NAC: N-acetylcysteine; SAR: Saroglitazar.

The current study revealed significant differences across all parameters between the model of cardiac toxicity group (5-FU) and the treated groups. In the 5-FU group, the congestion was 16.45%, which significantly decreased to 5.18% in the SAR (5 mg/kg)-treated group, (*p*-value < 0.01), while, the SAR (0.5 mg/kg)- and NAC (100 mg/kg)-treated groups showed 12.18% and 12.04%, respectively, (*p*-value < 0.05). Inflammatory cell infiltration in the 5-FU (150 mg/kg) group was 25.14%, decreasing to 12.92% in the SAR (5 mg/kg) group, (*p*-value < 0.01), while the SAR (0.5 mg/kg)- and NAC (100 mg/kg)-treated groups exhibited 20.28% and 19.50%, (*p*-value < 0.05). Endothelial cell loss in the 5-FU (150 mg/kg) group was 52%, significantly reduced to 21.23% in the SAR (5 mg/kg) group, (*p*-value < 0.01), with the low-dose SAR- and NAC (100 mg/kg)-treated groups showing 34.02% and 41.72%, (*p*-value < 0.05). Arterial wall thinning in the 5-FU (150 mg/kg) group was 52.32%, reduced to 15.71% in the SAR (5 mg/kg) group, (*p*-value < 0.01), while the SAR (0.5 mg/kg)- and NAC (100 mg/kg)-treated groups showed 45.70% and 48.79% with no significant differences compared to the 5-FU group. Degeneration in the 5-FU (150 mg/kg) group was 3.2, significantly reduced to 1.21 in the SAR (5 mg/kg) group, and with the SAR (0.5 mg/kg)- and NAC (100 mg/kg)-treated groups exhibiting 1.84 and 1.89, respectively (*p*-value < 0.05). By combining scores, the SAR (5 mg/kg) group demonstrated mild myocardial toxicity, while the SAR (0.5 mg/kg)- and NAC (100 mg/kg)-treated groups showed moderate toxicity. Statistical analysis revealed significant differences between the treated groups for all histopathological lesions, as quantified in Table 3 and illustrated in (Figure 5, Figure 6, Figure 7, Figure 8 and Figure 9).

## 4. Discussion

The multifaceted effects of PPAR agonists on the heart have gained significant attention recently, as they offer cardioprotective benefits through various mechanisms, including regulation of cardiac metabolism, endothelial function, inflammation, and oxidative stress [14,27]. The administration of 5-FU resulted in elevated levels of cardiac-specific biomarkers including CK-MB, troponin, BNP, and atherogenic indices. These biomarkers are highly indicative of cardiac injury in the context of drug-induced cardiac damage. Minor elevation in the level of these biomarkers is associated with a high risk of cardiac injury [28]. The use of SAR in the present study demonstrated a cardioprotective effect through attenuating the cardiac-specific biomarkers and atherogenic indices specifically AC and AIP compared to 5-FU and the well-known antioxidant NAC. These biomarkers are essential indicators for diagnosing and managing cardiac toxicity [29,30,31]. PPARα and PPARβ/δ are profusely expressed in heart tissues, and they are responsible for regulating genes involved in fatty acid uptake and oxidation. Activation of these receptors improves energy generation and maintains heart efficiency by increasing mitochondrial fatty acid β-oxidation. This metabolic flexibility has a pivotal role in preventing ischemic injury [32].

Atherosclerosis is a chronic process [33], whereas our model involves acute cardiotoxicity induced by a single high dose of 5-FU; however, atherogenic indices were measured in the current study to capture any early lipid-related changes that may contribute to the cardioprotective effects of SAR, even acutely. Additionally, 5-FU can acutely alter lipid metabolism, contributing to endothelial dysfunction and oxidative stress [34,35], which are relevant even in acute toxicity models. SAR has an important role in the regulation of lipid metabolism, attenuating both LDL and TG and increasing HDL. Additionally, it boosts fatty acid oxidation contributing to diminishing the risk of atherosclerosis and coronary artery disease. Additionally, SAR showed a noticeable attenuation in atherogenic lipoprotein in a study conducted on diabetic patients with dyslipidemia for twelve weeks [36]. PPAR-γ agonists have been shown to stabilize atherogenic plaques and attenuate the risk of rupture by inhibiting foam cell formation and smooth muscle cell migration, which have a pivotal role in atherosclerosis progression [37,38,39]. Other cardiac indices such as troponin-to-serum creatinine and BNP-to-troponin ratios have gained remarkable attention as new indicators of cardiac injury [40,41], and the use of SAR mitigated these ratios; however, it did not reach a significant level. Liver function biomarkers AST, ALT, ALP, and LDH, besides being assessed in clinical settings to evaluate liver damage and function, can also reflect cardiac injury. In the current study, the levels of these enzymes increased by the administration of 5-FU, and SAR successfully attenuated their level even more than NAC. These enzymes are present in many tissues, although they are not specific for heart injury; however, their elevation when interpreted in conjunction with other specific cardiac biomarkers such as troponin, BNP, and CK-MB may indicate cardiac toxicity [42].

In the current study, the use of 5-FU contributed to enhancing the expression of inflammatory cytokines that manifested through the high levels of ESR, CRP, TNF-α, and IL1β. Both low and high doses of SAR and NAC were able to ameliorate the inflammation with the maximum effect offered by the high dose of SAR. The ESR-to-CRP ratio has recently been involved in the diagnosis of cardiac diseases [43]; nevertheless, no significant alteration was noticed in the current study. SAR increases endothelial function and lowers pro-inflammatory indicators like C-reactive protein (CRP), both of which protect against vascular damage [44].

Additionally, PPAR agonists exert anti-inflammatory actions by inhibiting the expression of pro-inflammatory cytokines and adhesion molecules [45]. This reduction in inflammation mitigates myocardial damage and preserves cardiac function during stress conditions. For instance, PPARα activation has been shown to suppress inflammatory responses in cardiac tissues, contributing to its cardioprotective properties [46]. Furthermore, the activation of PPAR-γ was shown to ameliorate the inflammatory cytokines such as TNF-α, IL-6, and IL-1β formed by macrophages [47]. Moreover, a study revealed that by altering signaling pathways involving nuclear factor κ-light-chain-enhancer of activated B cells (NF-κB), activator protein 1 (AP-1), and signal transducer and activator of transcription proteins (STATs), PPAR agonists prevent the activation of genes linked to the inflammatory response, including IL-2, IL-6, IL-8, TNF-α, and metalloproteases [48]. Adipogenesis is also mediated by PPARγ activation; research has shown that natural PPARγ ligands like 15d-PGJ2 and synthetic ligands like rosiglitazone lower serum levels and TNF-α transcription [49,50,51].

Numerous studies have shown that the dual PPARα/γ agonist SAR has significant anti-inflammatory properties. In animal models of non-alcoholic steatohepatitis (NASH), SAR mitigated hepatic steatosis and fibrosis via a modulatory effect on inflammatory cytokines and adiponectin levels. In particular, it significantly improved liver inflammation and fibrotic lesions by efficiently lowering the expression of pro-inflammatory genes like TNF-α, IL-6, TGF-β, and monocyte chemoattractant protein-1 (MCP-1) [52]. Further research on hepatic stellate cells revealed that SAR exerts anti-fibrotic effects by modulating the TGF-β/Smad signaling pathway [53]. Additionally, a combination therapy of SAR and vitamin E has been proposed to improve the outcome of NASH by enhancing anti-inflammatory activity [54].

Oxidative damage is a key contributor to cardiac injury. Although TAOC and MDA levels were not measured directly in cardiac tissue, the detected amendments in the systemic levels of these oxidative damage markers can still offer valuable insight into the oxidative status correlated with cardiotoxicity. Systemic MDA is a well-established biomarker of lipid peroxidation, and elevated levels have been connected with oxidative damage in the heart and various other organs [55]. Likewise, decreased TAOC mirrors an overall reduction in antioxidant capacities, which is a characteristic of drug-induced cardiotoxicity. In tune with the current study, several studies have reported that systemic oxidative stress markers are reflective of cardiac oxidative damage [56,57]. Therefore, the systemic changes in TAOC and MDA observed in our study may indicate an underlying oxidative stress burden contributing to cardiotoxic outcomes. The low and high doses of SAR successfully boosted the systemic antioxidant capacity by increasing the blood level of TAOC which was parallel to that produced by NAC. Additionally, a high dose of SAR could also attenuate the production of MDA that is usually made from lipid peroxidation. Studies have shown that the activation of PPARβ/δ has been linked with enhanced defense mechanisms of the antioxidant system, reducing oxidative stress in myocardial cells. This effect helps in protecting mitochondrial function and inhibiting apoptosis, thereby maintaining cardiac integrity during ischemia–reperfusion events [58,59]. Furthermore, numerous studies have shown that SAR, a dual PPARα/γ agonist, has strong antioxidant benefits, as activating PPARα and PPARγ pathways enhances fatty acid oxidation which contributes to reducing lipotoxicity-induced oxidative stress via increasing the activity of nuclear factor erythroid 2-related factor 2 (Nrf-2). A crucial transcription factor called Nrf-2 controls the expression of genes that produce antioxidants, which lowers oxidative damage [60] In tune with the current study, treatment with SAR attenuated MDA levels and elevated the activity of antioxidant enzymes SOD and catalase in a study conducted on an epileptic model of rats [61]. Moreover, SAR was found to suppress the expression of NADPH oxidases in hepatic stellate cells, which contributed to the production of reactive oxygen species (ROS) and mitigated oxidative stress and cellular damage resulting from ROS via the inhibition of these enzymes [53].

Cardiac toxicity can significantly influence behavior in animal models. Studies have shown that rats with experimentally induced acute myocardial infarction exhibit decreased exploratory behavior and locomotor activity in the open field test. This hypolocomotion is indicative of increased anxiety and reduced motivation to explore novel environments [62]. Furthermore, infarcted rats display behaviors consistent with heightened anxiety, such as spending less time in the center of the open field and increased immobility. These behaviors suggest that cardiac injury may lead to emotional disturbances [63]. The behavioral assessment in the current study demonstrated that 5-FU markedly decreased locomotion, indicated by a reduction in the number of lines crossed, a characteristic of chemotherapy-induced fatigue and toxicity [64]. NAC did not significantly enhance locomotor activity, whereas SAR, at both low and high doses, significantly restored the movement patterns, suggesting its potential role in mitigating chemotherapy-induced toxicity. This improvement could be attributed to SAR’s ability to modulate neuroinflammation and oxidative stress, factors implicated in chemotherapy-induced behavioral deficits [65]. Likewise, both doses of SAR significantly enhanced rearing activity compared to 5-FU, a benefit not observed with NAC, further emphasizing its modulatory benefits.

Anxiety-related behavioral assessments, exemplified by the time spent in the center in the open field test, demonstrated that 5-FU significantly decreased the time spent in the center, indicative of increased anxiety levels. NAC did not yield a significant decrease in anxiety, while high-dose SAR markedly enhanced the duration spent in the center, indicating an anxiolytic effect. This corresponds with earlier research indicating that PPARγ activation may provide protection and alleviate anxiety through the control of neuroinflammatory pathways [66].

Concerning the total time of movement and total time spent in one square, 5-FU significantly reduced total movement time, representing severe motor impairment and anxiety-like behavior. These behavioral changes correspond with previous research demonstrating that 5-FU disturbs neurotransmitter balance and induces toxicity, resulting in locomotor deficits and cognitive impairments [64], while NAC resulted in a non-significant elevation in movement time, indicating considerable neuroprotection via its antioxidant characteristics; nonetheless, it was inadequate to fully mitigate 5-FU-induced anxiety completely [67]. In contrast, SAR, at both low and high doses, significantly enhanced locomotor activity, suggesting that PPARγ activation improves mitochondrial function and decreases oxidative stress, which improves movement performance. This corroborates previously reported findings that PPARγ agonists modulate inflammatory cytokines and oxidative stress to enhance mobility in models of chemotherapy-induced fatigue [65]. Moreover, 5-FU significantly elevated the duration spent in one square, indicating diminished exploratory behavior and exacerbated anxiety-related symptoms, consistent with studies identifying chemotherapy-induced disruptions in serotonergic and dopaminergic pathways as primary factors contributing to anxiety-like behavior [64]. While NAC provided partial protection, it was unable to fully reverse anxiety-related behaviors. SAR, however, markedly diminished the duration spent in one square, indicating an anxiolytic effect mediated by PPARγ’s capacity to inhibit pro-inflammatory cytokines like TNF-α and IL-6, which are implicated in neuroinflammation-related anxiety and cognitive impairment [67].

The histopathological findings greatly support the biochemical tests and assure the cardioprotective effects of SAR, particularly the high dose. Structural alterations in the heart that may result in malfunction are known as cardiac remodeling. PPARγ agonists have been shown to suppress pathological cardiac remodeling by altering the expression of genes linked to hypertrophy and fibrosis. After damage, this modulation aids in maintaining heart function and structure [68,69]. Additionally, endothelial dysfunction is enhanced by PPAR agonists via decreasing endothelial activation and increasing the bioavailability of nitric oxide. These outcomes support improved arterial relaxation and decreased vascular inflammation, both of which are advantageous for cardiovascular health in general [70].

Several factors contribute to the development of cardiotoxicity. For instance, advanced age is consistently associated with an increased risk, likely due to the progressive decline in physiological cardiac reserve over time [71]. Additionally, female sex has been identified in some studies as a potential risk factor, possibly related to differences in cardiac structure and the influence of sex hormones [72]. In this context, a key limitation of the present study is the exclusive use of male rats. While this choice was made to minimize hormonal variability, it inherently limits the generalizability of the findings to both sexes. Given that sex hormones can significantly influence cardiac physiology and drug responses, future investigations should include both male and female subjects to comprehensively assess potential sex-specific differences in the cardioprotective effects of saroglitazar.

## 5. Conclusions

In conclusion, through a combination of metabolic regulation, antioxidants, stabilization of atherosclerotic plaques and anti-inflammatory properties, cardiac remodeling modulation, and endothelial function enhancement, the dual PPAR agonist SAR provides cardioprotective effects and is considered a promising therapeutic agent for a variety of cardiovascular disorders to be tested in clinical trials for its pleiotropic properties. More studies are warranted to explore the molecular mechanisms of cardioprotection offered by SAR through gene and protein expression analysis, mitochondrial function assays, and cardiac apoptosis markers.

## Figures and Tables

**Table 1:** Evaluation of Histopathological Lesion Scores and Grades in Cardiac Tissue.

Lesions	Interpretation	Score	Grade
Congestion	Absence	0	NO
	5–10% congestion	1	Mild
	10–15% congestion	2	Moderate
	>15% congestion	3	Sever
Inflammation	Absence	0	NO
	5–15% inflammatory cells	1	Mild
	15–25% inflammatory cells	2	Moderate
	>25% inflammatory cells	3	Sever
Endothelial loss	Absence	0	NO
	<25% Endothelial loss	1	Mild
	25–50% Endothelial loss	2	Moderate
	>50% Endothelial loss	3	Sever
Arterial wall	Absence	0	NO
	<25% thinning	1	Mild
	25–50% thinning	2	Moderate
	>50% thinning	3	Sever
Degeneration	Absence	0	NO
	<1.5% degenerated area	1	Mild
	1.5–2% degenerated area	2	Moderate
	>3% degenerated area	3	Sever

**Table 2:** Impact of different doses of SAR on the change in total body weight, AST, ALT, LDH, and ALP.

Parameters	NC	5-FU	NAC	SAR 0.5	SAR 5
**Δ Weight**	42.14 ± 14.6	42.14 ± 15.7	27.14 ± 20	18.5 ± 16.7 *	32.86 ± 17.9
**AST**	113.7 ± 10.92	155.9 ± 19.1 ^###^	136.1 ± 8 *	133.1 ± 16.2 *	130.6 ± 9.5 *
**ALT**	47.23 ± 9.7	56.8 ± 3.1 ^#^	53.3 ± 8.5	56.8 ± 9.3	49 ± 4.3 **
**AST/ALT**	2.44 ± 0.5	2.85 ± 0.3	2.69 ± 0.47	2.37 ± 0.2 *	2.6 ± 0.2
**ALP**	267.8 ± 59	341 ± 27 ^#^	283.8 ± 58 *	248 ± 69 **	272 ± 35 **
**LDH**	273 ± 72	550 ± 96 ^###^	342 ± 29 **	429 ± 66	465 ± 89

Values are presented as mean ± S.D (n = 7). Based on one-way ANOVA followed by Tukey’s post hoc test, values with (#) indicate that 5-FU significantly differs from the NC group, (# *p*-value < 0.5, and ### *p*-value < 0.001); (*) indicates a statistically significant difference with the 5-FU group (* *p*-value < 0.05, and ** *p*-value < 0.01). NC: negative control, 5-FU: 5-Fluorouracil, NAC: N-acetylcysteine, SAR: Saroglitazar; AST: aspartate transaminase; ALT: alanine transaminase; LDH: Lactate dehydrogenase; ALP: alkaline phosphatase.

**Table 3:** Quantitative Analysis of Myocardial Toxicity.

Groups	Congestion	Inflammation	Endothelial Loss	Arterial Wall Thinning	Degeneration	Score	Grade
**NC**	0 ± 0.00	0 ± 0.00	0 ± 0.00	0 ± 0.00	00 ± 0.00	0	No Lesion
**5-FU (150 mg/kg)**	16.45 ± 4	25.14 ± 2.34	52.89 ± 1.5	52.32 ± 0.48	3.20 ± 0.95	3	Severe
**NAC (100 mg/kg)**	12.18 ± 1.46 *	20.28 ± 1.72 *	41.72 ± 2.21 *	48.79 ± 0.3	1.89 ± 0.14 *	2	Moderate
**SAR (0.5 mg/kg)**	12.04 ± 1.17 *	19.50 ± 1 *	34.02 ± 1.57 *	45.70 ± 0.48	1.84 ± 0.28 *	2	Moderate
**SAR (5 mg/kg)**	5.18 ± 1 **	12.92 ± 1.81 **	21.32 ± 2.82 **	15.71 ± 0.34 **	1.21 ± 0.43 *	1	Mild

Values are presented as mean ± S.D (n = 7). Based on one-way ANOVA followed by Tukey’s post hoc test, (*) indicates a statistically significant difference with the 5-FU group (* *p*-value < 0.05, and ** *p*-value < 0.01). NC: negative control, 5-FU: 5-Fluorouracil, NAC: N-acetylcysteine, SAR: Saroglitazar.

## Data Availability

Data from the current study are available on request.
